# Update on sex specific risk factors in cardiovascular disease

**DOI:** 10.3389/fcvm.2024.1352675

**Published:** 2024-02-06

**Authors:** Andrew H. Nguyen, Madelyn Hurwitz, Scott A. Sullivan, Antonio Saad, Jamie L. W. Kennedy, Garima Sharma

**Affiliations:** ^1^ Department of Medicine, Inova Fairfax Hospital, Falls Church, VA, United States; ^2^ School of Medicine, University of Virginia, Charlottesville, VA, United States; ^3^ Department of Maternal Fetal Medicine, Inova Fairfax Hospital, Falls Church, VA, United States; ^4^ Department of Cardiology, Inova Schar Heart and Vascular Institute, Falls Church, VA, United States

**Keywords:** women's cardiovascular health, obstetrics and gynecology, reproductive health, pregnancy, cardiovascular disease, sex-specific risk factors, adverse pregnancy outcomes, women's health

## Abstract

Cardiovascular disease (CVD) is the leading cause of death worldwide and accounts for roughly 1 in 5 deaths in the United States. Women in particular face significant disparities in their cardiovascular care when compared to men, both in the diagnosis and treatment of CVD. Sex differences exist in the prevalence and effect of cardiovascular risk factors. For example, women with history of traditional cardiovascular risk factors including hypertension, tobacco use, and diabetes carry a higher risk of major cardiovascular events and mortality when compared to men. These discrepancies in terms of the relative risk of CVD when traditional risk factors are present appear to explain some, but not all, of the observed differences among men and women. Sex-specific cardiovascular disease research—from identification, risk stratification, and treatment—has received increasing recognition in recent years, highlighting the current underestimated association between CVD and a woman's obstetric and reproductive history. In this comprehensive review, sex-specific risk factors unique to women including adverse pregnancy outcomes (APO), such as hypertensive disorders of pregnancy (HDP), gestational diabetes mellitus, preterm delivery, and newborn size for gestational age, as well as premature menarche, menopause and vasomotor symptoms, polycystic ovarian syndrome (PCOS), and infertility will be discussed in full detail and their association with CVD risk. Additional entities including spontaneous coronary artery dissection (SCAD), coronary microvascular disease (CMD), systemic autoimmune disorders, and mental and behavioral health will also be discussed in terms of their prevalence among women and their association with CVD. In this comprehensive review, we will also provide clinicians with a guide to address current knowledge gaps including implementation of a sex-specific patient questionnaire to allow for appropriate risk assessment, stratification, and prevention of CVD in women.

## 1 Introduction

Cardiovascular disease (CVD) is the leading cause of death in the United States among both men and women. Women in particular face significant disparities in their cardiovascular care when compared to men, both in the diagnosis and treatment of CVD (1–5). Even when traditional risk factors for CVD are present, clinicians are more likely to attribute a lower perceived risk in women leading to worse outcomes (1, 3, 5). For example, hypertension is more prevalent among women and carries a two-fold higher mortality risk compared to men (1, 6–9). Women with diabetes carry an excess risk of ischemic heart disease (IHD), and future risk of CVD by 3–7 fold vs. 2–3 fold compared to men (1, 10–14). Likewise, a recent meta-analysis demonstrated that tobacco use confers a 25% increased relative risk of major cardiovascular events in women when compared to men (1, 15). These discrepancies in the relative risk of CVD when conventional risk factors are present appear to explain some, but not all, of the observed differences among men and women.

Sex-specific risk factors and its association with CVD risk have become a highly researched field, stressing the importance of obtaining a thorough obstetric and reproductive history for cardiac risk stratification (1, 3, 5, 6, 8, 11, 16–18). Sex-specific risk factors including adverse pregnancy outcomes (e.g., hypertensive disorders of pregnancy, gestational diabetes, fetal growth restriction, preterm delivery, and placental abruption), premature menarche, premature menopause and vasomotor symptoms, polycystic ovarian syndrome, autoimmune disorders, infertility, and depression are all associated with increased future CVD risk. In fact, the American Heart Association (AHA)/American College of Cardiology (ACC) multi-society cholesterol guideline in 2018 and the AHA/ACC guideline on the primary prevention of CVD in 2019 identified “risk-enhancing factors” specific to women that are associated with increased incident atherosclerotic CVD risk (3). In this comprehensive review, we will cover each of these sex-specific risk factors in detail and their association with future CVD risk, heart failure (HF), and stroke. Additional entities including spontaneous coronary artery dissection (SCAD), coronary microvascular disease (CMD), systemic autoimmune disorders, and mental and behavioral health will be discussed in regards to their association with CVD. Additionally, we will provide strategies clinicians can utilize to incorporate a strong obstetric and reproductive history to better risk stratify for sex-specific CVD risk and directions for future research. Please note that we recognize patients have diverse gender identities and strive to use gender-inclusive language. In some instances throughout this review, we use the word “woman” (and the pronouns “she” and her”) to describe patients or individuals whose sex assigned at birth was female, whether they identify as female, male, or non-binary. When describing or referencing study populations used in prior research, we use the gender terminology reported by the study investigators.  

## 2 Data collection and analysis

Our comprehensive review used a structured systematic approach that included a methodical literature search of systematic peer-reviewed articles. We extracted data from landmark research between 1997 and 2023 from databases including PubMed, MEDLINE, EMBASE, Scopus, and the Cochrane Library. Keywords used in the selection of articles included terms referring to sex-specific risk factors in cardiovascular disease, adverse pregnancy outcomes, and hypertensive disorders of pregnancy. Our study has several limitations. First, our search was bound to certain inclusion criteria and a specific search strategy, which could have led to the non-inclusion of all relevant articles. Likewise, our search was limited to articles published in English; thus, perhaps not all relevant articles have been included. In addition, selection bias may have also affected our review. Lastly, the included articles are of different methodological quality, ranging from case reports to meta-analyses.

## 3 Sex specific risk factors

### 3.1 Adverse pregnancy outcomes

Pregnancy leads to metabolic, physiologic, and vascular changes in a mother which include insulin resistance, adipose deposition, hypercoagulability, cardiac remodeling, and decreased vascular resistance (19). Despite these necessary maternal adaptations to support fetal growth and development, the physiological stress of pregnancy can also cause adverse pregnancy outcomes (APOs) (19–24). APOs are common and occur in 17%–20% of all pregnancies in the US (16, 25–27), and are a constellation of interrelated maternal and fetal complications caused by incomplete placentation, oxidative stress, and/or vascular dysfunction (1, 19). The term encompasses disorders which will be discussed in detail under subparagraphs 3.1.1–3.1.5.

#### 3.1.1 Hypertensive disorders of pregnancy

Hypertensive disorders of pregnancy (HDP) are common complications during pregnancy and the early postpartum period. Pre-pregnancy chronic hypertension, gestational hypertension, preeclampsia, and eclampsia encompass the most common forms of HDP. Research across retrospective and prospective cohort studies have identified HDPs as a significant sex-specific risk factor for both short- and long-term maternal CVD (Table 1) (28–44). Women with history of HDP have significantly increased odds of chronic hypertension later in life (28, 29, 41, 42), stroke (30, 33, 34, 36, 39–41, 44), MI (44), and cardiomyopathy (44) versus women without history of HDP. Women with history of HDPs also have earlier-onset CVD and valvular heart disease including aortic stenosis and mitral regurgitation, suggesting an association between HDPs and accelerated cardiovascular aging (32, 84–86). Furthermore, women with HDPs are at highest risk for morbidity and mortality in the years following pregnancy compared to women without HDPs, including the development of cardiovascular risk factors such as hypertension, diabetes, and hyperlipidemia (32, 38, 84).

**Table 1:** A conglomerate of landmark studies describing statistically significant associations between various sex-specific risk factors and development of future cardiovascular risk factors, CVD, stroke, heart failure, and major adverse cardiac events.

Sex specific risk factor	Study	Study design	Study size/studies analyzed	Outcome of study/effect estimate (95% CI)
Hypertensive disorders of pregnancy (HDPs)	Behrens et al. (28)	Retrospective cohort	1.02 million	- HTN within first year postpartum: - Ages 20–29 [HR 11.6 (10.4–12.8)] - Ages 40–49 [HR 24.5 (21.8–27.6)]
	Grandi et al. (29)	Retrospective cohort	146,748	- CVD [HR 2.2 (1.7–2.7)] - HTN [HR 5.6 (5.1–6.3)]
	Theilen et al. (30)	Retrospective cohort	57,384	Recurrent HDP: - All-cause mortality [HR 2.04 (1.76–2.36)] - T2DM [HR 4.33 (2.21–8.47)] - IHD [HR 3.30 (2.02–5.40)] - Stroke [HR 5.10 (2.62–9.92)]
	Riise et al. (31)	Prospective cohort	20,075	- CVD [HR 1.5 (1.2–1.8)]
	Honigberg et al. (32)	Prospective cohort	220,024	- CAD [HR 1.8 (1.3–2.6)] - HF [HR 1.7 (1.04–2.6)] - AS [HR 2.9 (1.5–5.4)] - MR [HR 5.0 (1.5–17.1)]
Gestational hypertension	Heida et al. (33)	Meta-analysis	5 studies	- Overall CVD [RR 1.89 (1.31–2.72)] - IHD [RR 1.44 (1.30–1.60)] - Stroke [RR 1.41 (1.20–1.65)]
	Grandi et al. (34)	Meta-analysis	9 studies	- CVD [OR 1.67 (1.28–2.19)] - Stroke [OR 1.83 (1.79–4.22)]
	Haug et al. (35)	Prospective cohort	23,885	- CVD, ages 40–70 [HR 1.57 (1.32–1.87)]
	Canoy et al. (36)	Prospective cohort	1.1 million	All women with hypertensive pregnancies: - CHD [RR 1.29 (1.27–1.31)] - Ischemic stroke [RR 1.29 (1.23–1.35)] - Hemorrhagic stroke [RR 1.14 (1.07–1.21)] Women with hypertensive pregnancy, not taking HTN treatment at baseline: - CHD [RR 1.17 (1.14–1.19)] - Ischemic stroke [RR 1.18 (1.11–1.25)] - Hemorrhagic stroke [RR 1.09 (1.02–1.18)]
	Riise et al. (37)	Prospective cohort	617,589	- CVD [HR 1.8 (1.7–2.0)]
	Stuart et al. (38)	Prospective cohort	58,671	- HTN [HR 2.8 (2.6–3.0)] - T2DM [HR 1.7 (1.4–1.9)] - HLD [HR 1.4 (1.3–1.5)]
Preeclampsia	Wu et al. (39)	Meta-analysis	>6.4 million/22 studies	- HF [RR 4.19 (2.09–8.38)] - CHD [RR 2.50 (1.43–4.37)] - CV Death [RR 2.21 (1.83–2.66)] - Stroke [RR 1.81 (1.29–2.55)]
	Heida et al. (33)	Meta-analysis	12 studies	- CVD RR 2.15 (1.76–2.61) - IHD [RR 2.06 (1.68–2.52)] - Stroke [RR 1.53 (1.21–1.92)]
	Brown et al. (40)	Meta-analysis	43 studies	- HTN [RR 3.13 (2.51–3.89)] - CVD [OR 2.28 (1.87–2.78)] - Stroke [OR 1.76 (1.43–2.21)]
	Grandi et al. (34)	Meta-analysis	16 studies	- Moderate preeclampsia: CVD [OR 2.24 (1.72–2.93)] - Severe preeclampsia: CVD (OR 2.74 [2.48–3.04] - CVD mortality [OR 1.73 (1.46–2.06)] - Stroke [OR 2.95 (1.10–7.90)] - IHD [OR 1.73 (1.46–2.06)]
	Bellamy et al. (41)	Meta-analysis	3,488,160/25 studies	- HTN [RR 3.70 (2.70–5.05)] - IHD [RR 2.16 (1.86–2.52)] - Stroke [RR 1.81 (1.45–2.27)]
	Brouwers et al. (42)	Meta-analysis	22 studies	Recurrent preeclampsia: - HTN [RR 2.3 (1.9–2.9)] - IHD [RR 2.4 (2.2–2.7)] - HF [RR 2.9 (2.3–3.7)] - CVD hospitalization [RR 1.6 (1.3–1.9)]
	Riise et al. (43)	Prospective cohort	506,350	MACE [HR 2.1 (1.73–2.65)]
	Stuart et al. (38)	Prospective cohort	58,671	- HTN [HR 2.2 (2.1–2.3)] - T2DM [HR 1.8 (1.6–1.9)] - HLD [HR 1.3 (1.3–1.4)]
	Wu et al. (44)	Retrospective cohort	>44 million	- Stroke [OR 7.83 (6.25–9.80)] - MI [OR 5.20 (3.11–8.69)] - Peripartum cardiomyopathy [OR 4.37 (3.64–5.26)]
Gestational diabetes mellitus (GDM)	Bellamy et al. (45)	Meta-analysis	675,455/20 studies	- T2DM [RR 7.43 (4.79–11.51)]
	Vounzoulaki et al. (46)	Meta-analysis	1.33 million/20 studies	- T2DM [RR 9.51 (7.14–12.67)]
	Kramer et al. (47)	Meta-analysis	5.39 million/9 studies	- Overall future CV events [RR 1.98 (1.57–2.5)] - Future CV events restricted to women with GDM who did not develop T2DM [RR 1.56 (1.04–2.32)] - Future CV events in first decade postpartum [RR 2.31 (1.57–3.39)]
	Grandi et al. (34)	Meta-analysis	8 studies	- CVD [OR 1.68 (1.11–2.52)]
	Heida et al. (48)	Prospective cohort	22,265	- T2DM [HR 3.68 (2.77–4.90)]
	Kaul et al. (49)	Retrospective cohort	240,083	GDM only: - T2DM [HR 20.3 (18.1–22.6)] - HTN [HR 2.0 (1.8–2.2)] - CVD [HR 1.4 (1.0–1.9)] GDM and overweight: - T2DM [HR 40.1 (34.4–46.6)] - HTN [HR 3.7 (3.2–4.3)] - CVD [HR 2.1 (1.1–3.5)]
	Carr et al. (50)	Cross-sectional	995	Women with family history of T2DM: - HTN [OR 1.88 (1.34–2.64)] - HLD [OR 1.76 (1.28–2.44)]
Preterm delivery (PTD)	Wu et al. (51)	Meta-analysis	>5.8 million/21 studies	- CVD [RR 1.43 (1.18–1.72)] - CV mortality [RR 1.78 (1.42–2.21)] - CAD [RR 1.49 (1.38–1.60)] - Stroke [RR 1.65 (1.51–1.79)]
	Grandi et al. (34)	Meta-analysis	14 studies	- CVD [OR 1.6 (1.4–1.9)]
	Tanz et al. (52)	Prospective cohort	70,182	- CVD [HR 1.42 (1.16–1.72)]
	Tanz et al. (53)	Prospective cohort	57,904	- HTN [HR 1.11 (1.06–1.17)] - T2DM [HR 1.17 (1.03–1.33)] - HLD [HR 1.07 (1.03–1.11)]
	Parikh et al. (54)	Retrospective cohort	15,896	- HTN [OR 1.57 (1.04–2.37)]
Placental abruption/placental syndromes	Grandi et al. (34)	Meta-analysis	28.99 million/7 studies	- CVD [OR 1.8 (1.4–2.3)]
	Ray et al. (55)	Retrospective cohort	1.03 million	- CVD [HR 2.0 (1.7–2.2)]
Pregnancy loss	Oliver-Williams (56)	Meta-analysis	649,965/10 studies	Miscarriage: - CHD [OR 1.45 (1.18–1.78)] - Stroke [OR 1.11 (0.72–1.69)] Recurrent miscarriage: - CHD [OR 1.99 (1.13–3.50)]
	Hall et al. (57)	Prospective cohort	79,121	- CVD [HR 1.11 (1.06–1.16)]
	Ranthe et al. (58)	Prospective cohort	1.03 million	- MI [RR 1.13 (1.03–1.24)] - Ischemic stroke [RR 1.16 (1.07–1.25)] - HTN [RR 1.20 (1.05–1.38)]
	Smith et al. (59)	Retrospective cohort	129,290	- 1–2 loss, IHD [HR 1.48 (1.09–2.02)] - >1 loss, IHD [HR 1.52 (1.13–2.06)] - >3 loss, IHD [HR 2.35 (0.87–6.36)]
	Wagner et al. (60)	Retrospective cohort	60,105	- 2 + loss, IHD [HR 1.74 (1.22–2.52)] - 3 + loss, IHD [HR 3.18 (1.49–6.80)]
Stillbirth	Grandi et al. (34)	Meta-analysis	8 studies	- CVD [OR 1.5 (1.1–2.1)]
	Peters et al. (61)	Prospective cohort	>500,000	- CVD [HR 1.14 (1.02–1.28)]
	Ranthe et al. (58)	Prospective cohort	1.03 million	- MI [RR 2.69 (2.06–3.50)] - Ischemic stroke [RR 1.74 (1.32–2.28)] - HTN [RR 2.42 (1.59–3.69)]
Small for gestational age (SGA)	Heida et al. (33)	Meta-analysis	9 studies	- Overall CVD [RR 1.66, (1.26–2.18)] - IHD [RR 1.68, (1.31–2.14)] - Stroke [RR 1.62, (1.51–1.74)]
	Ngo et al. (62)	Retrospective cohort	812,732	- Moderate SGA, CVD [HR 1.36 (1.23–1.49)] - Severe SGA, CVD [HR 1.66 (1.47–1.87)]
	Bonamy et al. (23)	Retrospective cohort	923,686	- Moderate SGA, CVD [HR 1.39 (1.22–1.58)] - Severe SGA, CVD [HR 2.57 (1.97–3.34)]
Large for gestational age (LGA)	Morken et al. (63)	Prospective cohort	711,726	- CV mortality [HR 3.0 (2.0–4.6)]
Premature menarche	Charalampopoulos et al. (64)	Meta-analysis	9 studies	- All-cause mortality [HR 1.23 (1.10–1.38)]
	Lee et al. (65)	Prospective cohort	648	- MACE [RR 4.53 (2.13–9.63)]
	Canoy et al. (66)	Prospective cohort	1.2 million	- CHD [RR 1.27 (1.22–1.31)]
	Ley et al. (67)	Prospective cohort	73,814	- CVD [RR 1.22 (1.09–1.36)]
	Lakshman et al. (68)	Prospective cohort	15,807	- HTN [HR 1.13 (1.02–1.24)] - CVD [HR 1.17 (1.07–1.27)] - CHD [HR 1.23 (1.06–1.43)] - CVD mortality [HR 1.28 (1.02–1.62)]
	Peters and Woodward (61)	Prospective cohort	>500,000	- CVD [HR 1.10 (1.01–1.30)]
Polycystic ovarian syndrome (PCOS)	Amiri et al. (69)	Meta-analysis	30 studies	Women of reproductive age: - HTN [RR 1.70 (1.43–2.07)]
	Okoth et al. (70)	Meta-analysis	32 studies	- Overall CVD [OR 1.30 (1.09–1.56)] - CHD [OR 1.40 (1.13–1.84)] - Stroke [OR 1.36 (1.09–1.70)]
	Zhang et al. (71)	Meta-analysis	166,682/10 studies	- Overall CVD [OR 1.66 (1.32–2.08)] - MI [OR 2.57 (1.37–4.82)] - IHD [OR 2.77 (2.12–3.61)]—Stroke [OR 1.96 (1.56–2.47)]
Premature menopause	Muka et al. (72)	Meta-analysis	310,329/32 studies	- Overall CHD [RR 1.50 (1.28–1.76)] - Fatal CHD [RR 1.11 (1.03–1.20)]
	Ley et al. (67)	Prospective cohort	73,814	- CVD [RR 1.32 (1.16–1.51)]
	Honigberg et al. (73)	Prospective cohort	144,260	- Premature natural menopausal, CVD [HR 1.36 (1.19–1.56)] - Premature surgical menopause, CVD [HR 1.87 (1.36–2.58)]
Premature ovarian failure (POF)	Roeters et al. (74)	Meta-analysis	190,588/10 studies	- CVD [HR 1.61 (1.22–2.12)] - IHD [HR 1.69 (1.29–2.21)]
Infertility	Parikh et al. (75)	Prospective cohort	863,324	- CVD [HR 1.19 (1.02–1.39)]
	Magnus et al. (76)	Prospective cohort	64,064	- CVD [HR 1.14 (1.08–1.20)]
	Farland et al. (77)	Prospective cohort	103,729	- Overall CHD [HR 1.13 (1.01–1.26)] - Infertility ≤25 years: CHD [HR 1.26 (1.09–1.46)] - Infertility 26–30 years: CHD [HR 1.08 (0.93–1.25)] - Infertility >30 years: CHD [HR 0.91 (0.70–1.19)] - Infertility due to ovulatory disorder: CHD [HR 1.28 (1.05–1.55)] - Infertility due to endometriosis: CHD [HR 1.42 (1.09–1.85)]
In-vitro fertilization (IVF)	Dayan et al. (78)	Meta-analysis	1.44 million/6 studies	- CVD [HR 0.91 (0.67–1.25)] - T2DM [HR 0.93 (0.87–1.00)]
	Udell et al. (79)	Prospective cohort	1.19 million	- CVD [HR 0.55 (0.41–0.74)]
Systemic erythematous lupus (SLE)	Li et al. (80)	Meta-analysis	9 studies	- All genders: CVD [RR 3.39 (2.15–5.35)] - Women: CVD [RR 3.27 (2.01–5.30)] - Men: CVD [RR 3.16 (2.02–4.94)]
	Manzi et al. (81)	Retrospective cohort	2,706	- Women age 35–44, MI [RR 52.43 (21.6–98.5)]
Rheumatoid arthritis (RA)	Aviña-Zubieta et al. (82)	Meta-analysis	111,758/24 studies	- CV death [RR 1.50 (1.39–1.61)]
Depression	Rosengren et al. (83)	Case-control	24,767	- MI [AR 9% (7–10)]

HTN, hypertension; CAD, coronary artery disease; HF, heart failure; AS, aortic stenosis; MR, mitral regurgitation; CVD, cardiovascular disease; CV, cardiovascular; T2DM, type II diabetes mellitus; IHD, ischemic heart disease; CHD, coronary heart disease; HLD, hyperlipidemia; MACE, major adverse cardiac events; MI, myocardial infarction.

Gestational hypertension is defined as pregnancy-induced hypertension (defined as SBP ≥ 140 mmHg or DBP ≥ 90 mmHg) after 20 weeks gestation without evidence of proteinuria or preeclampsia (3, 19). History of gestational hypertension has been consistently associated with increased CVD risk and increased odds of stroke across various studies (Table 1) (33–38).

Among the major types of HDPs, preeclampsia poses the greatest morbidity and mortality risk and affects 5%–10% of all pregnant women (16, 87–89). Preeclampsia is a condition in which preexisting or new-onset hypertension is complicated by proteinuria and/or other features of end-organ dysfunction after 20 weeks gestation (16). There is robust research to suggest that history of preeclampsia is independently associated with increased risk of CVD, IHD, stroke, and chronic hypertension later in life (Table 1) (33, 34, 38–44). For example, a meta-analysis by Wu et al. of 6.4 million women demonstrated a 4-fold increased risk of IHD and 2-fold increased risk of HF in women studied with preeclampsia compared to those without. Of note, women with recurrent preeclampsia compared to women with an isolated episode of preeclampsia are at significantly higher risk for future CVD (37, 84, 90), hypertension, and IHD (16, 30, 42). Despite the research demonstrating an independent association between preeclampsia and CVD, attempts to incorporate preeclampsia within risk scoring equations have led to only small improvements in discrimination and reclassification (91). This may be in part due to the population-based cohort studies including women well beyond their reproductive years rather than those of childbearing age (91, 92). Future studies should work to incorporate women closer to the target population intended for CVD screening and preventative intervention (6, 91).

#### 3.1.2 Gestational diabetes Mellitus

Gestational diabetes mellitus (GDM) is a condition of impaired glucose tolerance during pregnancy that most commonly develops during the second and third trimester (16, 93). Paralleling the rise in prevalence of obesity, GDM has become increasingly prevalent, now estimated to affect 6%–9% of all pregnant women in the US (16, 94, 95). GDM results from inadequate response from pancreatic beta-cells to respond to the physiological and placental-mediated insulin resistance which occurs during pregnancy (84, 96). Several meta-analyses have shown that women with GDM are at increased risk of developing cardiovascular risk factors including type 2 diabetes mellitus (T2DM), hypertension, and hyperlipidemia leading to early-onset CVD, future cardiovascular events, and fatal IHD (Table 1) (34, 45–50). In fact, women with GDM have a 7- to 10-fold increased risk of developing T2DM (16, 45, 84) and nearly a 2-fold increased risk of developing hypertension and hyperlipidemia (Table 1) (16, 46, 49, 50, 97). This relative risk for future CVD remained statistically significant even after restricting the sensitivity analysis to women with GDM who did not subsequently develop T2DM (47). Proposed mechanisms to explain the association between GDM and early-onset CVD include epigenetics, elevated inflammatory markers including CRP and IL-6 associated with early atherosclerosis, and endothelial dysfunction leading to subsequent increased carotid artery thickness (16, 98). Some researchers suggest a dose-dependent relationship between the degree of glucose impairment during pregnancy with risk of subsequent CVD (84, 99). Nonetheless, documenting an obstetrical history of GDM in women is crucial given these associations with CVD which have been demonstrated consistently throughout studies (Table 1) (84, 99).

#### 3.1.3 Preterm delivery

Spontaneous preterm delivery (sPTD), defined as a live birth before 37 weeks gestation, is a significant cause of neonatal mortality worldwide (16). Although our understanding of the underlying mechanism is limited, sPTD is associated with an increased development of cardiovascular risk factors and maternal CVD mortality (3, 52, 53, 84, 100, 101). For example, in the first decade after pregnancy, women with a history of sPTD are at increased risk of developing chronic hypertension, T2DM, hypercholesterolemia, and subclinical atherosclerosis (Table 1) (34, 51–53, 101). A meta-analysis by Wu et al. highlighted the association of sPTD with increased risk of future composite CVD, cardiovascular mortality, CAD, and stroke (Table 1) (51). Emerging research now suggests that the earlier sPTD occurs in pregnancy, the stronger its association with later development of hypertension and increased maternal CVD risk (3, 19, 52, 54, 84, 102).

#### 3.1.4 Placental abruption and pregnancy loss

Placental abruption is defined as the premature separation of a normally implanted placenta from the uterus before delivery most often occurring in the third trimester, and is strongly associated with cardiovascular risk factors and increased maternal CVD risk (3, 55, 84, 103). A meta-analysis by Grandi et al. demonstrated an increased risk of CVD in women with history of placental abruption (34), findings similarly documented in a large retrospective study by Ray et al., reporting a 1.7-fold risk of CVD in women with history of placental abruption or infarction (Table 1) (55). There is also a strong association of placental abruption with other concomitant APOs and cardiovascular risk factors such as higher BMI, hyperglycemia, and hyperlipidemia (84, 103).

Likewise, all forms of pregnancy loss (miscarriage, stillbirths, or combined) are associated with elevated risk of future cardiovascular risk factors and major cardiovascular events later in life (Table 1) (56–59, 61, 104). Recurrent pregnancy loss, defined as 3 or more losses, are associated with a particularly increased CVD risk (3). For example, a study by Wagner et al. demonstrated a higher risk of CVD for women who experienced two or three or more miscarriages as compared to those who did not experience miscarriage (Table 1) (60). Outcome data studying conventional CVD risk factors indicate that miscarriage is independently associated with future CVD and MI, highlighting its importance in obstetrical history for cardiovascular risk stratification in women (56, 59, 84).

#### 3.1.5 Small for gestational age (SGA) and large for gestational age (LGA)

The association between infant birth weight and future maternal CVD risk is well documented in current literature though studies are limited, thus warranting future investigation (Table 1) (23, 33, 62, 63). For example, in the Women’s Health Initiative, delivery of a small for gestational age (SGA) infant (defined as being ≤10th percentile in weight for their gestational age) was independently associated with increased maternal ASCVD risk after adjustment for conventional cardiovascular risk factors (19, 84, 105). A retrospective cohort study by Bonamy et al. observed similar findings, reporting a 3-fold maternal CVD risk in women with preterm or SGA infants even after accounting for pregnancy-related complications, socioeconomic factors, and tobacco use (23). This complex interplay between fetal growth restriction (FGR) and maternal CVD risk is hypothesized to be related to maternal vascular health (19). Many cases of FGR are thought to result from uteroplacental insufficiency due to poor implantation of the spiral arteries, or vascular insufficiency due to abnormal maternal uterine artery flow resulting in inadequate oxygen and nutrient supply to the fetus (19, 84). Thus, delivery of a SGA infant may unmask preexisting maternal vascular dysfunction which can result in a future increased predisposition for CVD including HF and stroke (19).

A need for further research is warranted in mothers who deliver infants large for gestational age (LGA), defined as an infant whose weight is ≥90th percentile for their gestational age, as emerging studies suggest that LGA delivery may be related to increased CVD risk—possibly mediated by its association with elevated BMI and diabetes (Table 1) (19, 63, 84, 106, 107).

### 3.2 Premature menarche

Premature menarche, defined as menarche occurring before age 12, is strongly associated with an increased risk for developing future cardiovascular risk factors and CVD (3, 84). Though the mechanism linking early menarche to increased CVD risk is not entirely understood, it is postulated that given the strong association between childhood BMI and early menarche, premature menarche may reflect both genetic (e.g., elevated leptin levels associated with increased adiposity and higher BMI) and lifestyle risk factors (e.g., excess calorie consumption, lower birth weight, reduced physical activity) (84, 108, 109). One study estimated that premature menarche, independent of sociodemographic factors, is associated with a 15%–30% increased risk of future CVD (Table 1) (61, 64–66, 68). A meta-analysis by Charalampopoulos et al. reported a 3% reduction in the relative risk of all-cause mortality for every 1-year increase at menarche, and those women who experienced menarche at age <12 vs. ≥12 years were at an increased risk of all-cause mortality (Table 1) (64). The strong association intertwining premature menarche and increased CVD risk is likely due to women with history of early menarche being more susceptible to developing shared risk factors including hypertension, T2DM, hypercholesterolemia, and obesity later in life (3, 66, 68, 84).

Emerging data now suggest the relative risk for future CVD is elevated in both premature and delayed menarche, defined as menarche age ≥17 years, though further research is needed (1, 61, 65, 66, 84).

### 3.3 Polycystic ovarian syndrome (PCOS)

Polycystic ovarian syndrome (PCOS) is the most common cause of infertility in women and is often diagnosed in adolescence with key features including hyperandrogenism, ovulatory dysfunction, and polycystic kidneys on imaging (3, 84, 110). Women with PCOS are more likely to have traditional CVD risk factors including hypertension, insulin resistance, metabolic syndrome, elevated BMI, and dyslipidemia (1, 3, 69, 84, 110, 111). A meta-analysis by Zhang et al. demonstrated that the pooled risk of CVD events was higher in women with PCOS when compared to non-PCOS women, including increased risk of MI, IHD, and stroke (Table 1) (71). Likewise, a recent meta-analysis by Okoth et al. found that PCOS was associated with a 30% higher risk of overall CVD, including both in the risk of HF and stroke (Table 1) (70). These notable associations may be explained by the relationship between PCOS and carotid intima-media thickness (CIMT) and coronary artery calcium (CAC). Women with PCOS have greater CIMT and CAC even after adjusting for BMI when compared to non-PCOS women (3, 84, 112–115).

### 3.4 Premature menopause, premature ovarian failure, and vasomotor symptoms

Premature menopause is commonly defined as the permanent cessation of menses before the age of 40 and is often attributed to premature ovarian failure (POF). POF, a condition characterized by hypergonadotropic hypogonadism, exhibits symptoms from hypoestrogenism including amenorrhea, hot flashes, and vaginal dryness. A shorter reproductive lifespan and an earlier age at menopause transition (MT) mediated by hypoestrogenism has been well-studied as an independent risk factor for CVD (3, 116). Estrogen assists in blood flow regulation and the relaxation of blood vessels, and in tandem with early loss of ovarian function can lead to long-term activation of the renin-angiotensin-aldosterone system, chronic inflammation, and vascular damage (3, 117). Hypoestrogenism also leads to dysfunction in cholesterol metabolism leading to atherosclerotic plaque formation and an elevated testosterone-to-estradiol ratio, factors which can increase subsequent risk of CVD and HF (3, 118).As such, a recent scientific statement by the AHA identified the MT as a particularly impactful period requiring an aggressive prevention-based approach for women to prevent accelerated CVD risk and future cardiovascular events (84, 119).

Vasomotor symptoms (VMS), including night sweats, hot flashes, and heat intolerance, are the hallmarks of the MT and can significantly impact quality of life (120–123). Emerging studies show evidence of an association between VMS with aortic calcification (124) and increased odds of elevated BMI, total cholesterol, and hypertension (125).

Premature menopause and POF have been consistently associated with greater maternal CVD and mortality risk across high-quality data studies cited in this review, as noted in Table 1 (67, 72–74, 84). For example, a meta-analysis by Muka et al. assessed the relationship between premature menopause and CVD among 190,588 women, demonstrating an increased risk of overall incident CVD and CVD mortality (72).

### 3.5 Infertility/in-vitro fertilization (IVF)

Women with a history of infertility, defined as the inability to achieve pregnancy after ≥12 months of unprotected intercourse, excluding causes of male infertility, have a higher prevalence of conventional CVD risk factors and a strong association with CVD (79, 84, 126, 127). The largest study to date using Swedish registry data analyzed 863,324 participants, reporting a 19% greater risk of CVD in women who experienced ≥5 years of infertility versus women who did not experience infertility (75). This significant association between infertility and CVD was consistent in both age-adjusted and multivariable adjusted models across other large prospective cohort studies (Table 1) (75–77). The risk of CVD appears to be the strongest among women with history of infertility at an earlier age and among women whose infertility is attributable to an ovulatory disorder or endometriosis (77). Further research is necessary, however, to identify infertility as an independent risk factor for CVD as there are many shared risk factors and comorbidities (84).

Emerging research has also shown that the use of assisted reproductive technology (ART), including *in vitro* fertilization (IVF) and intracytoplasmic sperm injection, are associated with increased CVD risk (Table 1) (79, 126). This may be due to a causal relationship between ART and APOs, as one systematic review reported an association between IVF and HDPs (79, 126), though further research regarding the long-term cardiovascular implications of ART is needed.

### 3.6 Spontaneous coronary artery dissection and coronary microvascular disease

Spontaneous coronary artery dissection (SCAD) is an acute coronary event related to development of a hematoma within the tunica media causing separation of the intima or intima-media complex from the underlying vessel and compression of the true lumen, leading to ischemia and acute MI (128). Two hypotheses have been postulated to describe the pathophysiology of SCAD: the “inside-out” hypothesis and the “outside-in” hypothesis (128–130). The “inside-out” hypothesis suggests that blood enters the subintimal space from the true lumen after an endothelial-intimal disruption, while the “outside-in” hypothesis suggests that a hematoma arises *de novo* in the media perhaps from disruption of traversing microvessels (128–130). Current evidence favors the “outside-in” hypothesis for three reasons: (1) most SCAD cases demonstrate no communication between false and true lumens (128, 129, 131, 132); (2) serial angiograms following a SCAD event demonstrate that development of an intramural hematoma precedes intimal dissection (128, 129); and (3) optical coherence tomography (OCT) imaging suggests that observed fenestrations may arise from rupture of the false lumen into the true lumen, rather than vice versa (128, 132). Strikingly, women comprise 87%–95% of all SCAD events with literature describing SCAD as the underlying cause of up to 35% of all acute coronary syndrome cases in women ≤50 years of age and is the most common cause of pregnancy-associated MI (128, 133–138). The explanation for the astonishing over-representation of SCAD in women remains a hot topic for debate as many of the current leading theories have conflicting results and are not fully understood. Several postulated triggers for SCAD include but are not limited to: (1) genetic underpinnings; (2) regulation of autosomal susceptibility genes that exhibit sex-specific regulation (e.g., estrogen response element genes); (3) intrinsic, gene-independent differences in coronary biology in women; (4) endogenous and exogeneous sex hormones; and (5) extreme physical or emotional stress (128, 135, 139–141).

Aforementioned, pregnancy-associated SCAD (P-SCAD) is the most common cause of pregnancy-associated MI, estimated to affect 1.81 per 100,000 pregnancies and comprises 14.5%–43% of all pregnancy-associated MI events (128, 142–144). The majority of P-SCAD events occur in the third trimester or early postpartum, and when compared to non-P-SCAD women, these patients tend to be older at first childbirth with more severe clinical presentation (e.g., impaired left ventricular function, cardiogenic shock, left main disease, and multivessel dissections) (128, 145–148). The cause of P-SCAD is not fully understood, however hormonal changes during pregnancy leading to deleterious alterations in the architecture of the arterial wall has been hypothesized (138). Nonetheless, given the unpredictable and recurrent nature of SCAD, women are often advised to avoid subsequent pregnancy following an acute SCAD event (128). It should be highlighted that patients with SCAD experience a high frequency of major adverse cardiovascular events (MACE) driven primarily by recurrent SCAD, with rates of SCAD recurrence ranging from 10 to 30% by varying reports (128). Additionally, all patients diagnosed with SCAD should be assessed for other concomitant arterial abnormalities, given its high association with aneurysmal disease and fibromuscular dysplasia (138, 149–151).

There is now greater recognition and appreciation of the impact of structural and functional disorders that affect the entire coronary circulation, including microcirculation, termed coronary microvascular disease (CMD) (152, 153). Conceptually, the coronary arterial system can be divided into three compartments: (1) epicardial coronary arteries; (2) pre-arterioles; and (3) intramyocardial arterioles (152). Together, the pre-arterioles and intramyocardial arterioles directly interface with the capillary bed and comprise the microcirculation (152). In the absence of obstructive stenosis, the larger epicardial coronary arteries contribute only 10% of the coronary circulation volume, while the microcirculation contributes the remaining 90% and thus, is the site of the majority of coronary blood flow resistance and its regulation (152). The interconnected regulatory pathways which allow for dynamic regulation of microcirculatory resistance to match myocardial oxygen consumption is disrupted in CMD through a combination of structural (e.g., luminal narrowing, intramyocardial or perivascular fibrosis, decreased capillary density) and functional abnormalities (e.g., impaired endothelial dilation, microvascular spasm, enhanced constrictive reactivity), resulting in ischemia and a constellation of symptoms (152–154).

A proposed CMD classification scheme include the following subtypes: (1) primary CMD with evidence of ischemia with no obstructive CAD (INOCA); (2) CMD in MI with non-obstructive CAD (MINOCA) (3) CMD with obstructive CAD post-MI; (4) iatrogenic CMD associated with reperfusion injury and microvascular distal embolization following coronary revascularization; and (5) CMD unrelated to atherosclerosis (152–154). By far the most prevalent presentation of CMD occurs in patients with signs and symptoms of INOCA, seen most particularly in women (152). For example, in both the WISE (Women's Ischemia Syndrome Evaluation) and WISE-CVD (Women's Ischemia Syndrome Evaluation—Coronary Vascular Dysfunction) studies, nearly half of women with INOCA had CMD detected by invasive testing (152, 155, 156). Likewise, particularly in women, CMD is a major driver for adverse CV death and hospitalization for MI and HF (152, 157, 158). CMD is therefore an important and underrecognized entity to understand when observing similar or worse outcomes for women with INOCA despite a lower rate of obstructive epicardial CAD (152). Cardinal manifestations include angina, exertional dyspnea, and HF symptoms and when present without explanatory obstructive CAD, should prompt further diagnostic testing for CMD (152, 153). In the 2021 AHA/ACC/ASE/CHEST/SAEM/SCCT/SCMR Guideline for the Evaluation and Diagnosis of Chest Pain, evaluation for CMD with invasive coronary function testing and non-invasive assessment of myocardial blood flow by positron emission tomography (PET), stress cardiac magnetic resonance (CMR) imaging, and stress echocardiography with coronary flow velocity reserve was provided a class 2a recommendation for patients with stable angina and evidence of non-obstructive CAD (152, 154, 159). Given the paucity of robust evidence from large-scale randomized trials, there are no existing management guidelines for CMD (152). Treatment is aimed at reducing risk of adverse CV events and treating symptoms targeted to the specific subtype of CMD (152). The emerging WARRIOR (Women's Ischemia Trial to Reduce Events in Non-Obstructive CAD) trial will provide important outcome data at 3-year follow-up on the impact of medical therapy MACE in women with symptoms of INOCA, a population with a high rate of CMD (152, 160).

### 3.7 Systemic autoimmune and autoimmune disorders

Systemic inflammatory and autoimmune disorders, such as systemic erythematous lupus (SLE), rheumatoid arthritis (RA), and psoriasis are more prevalent in women and have shown clear association with increased MI and CVD mortality risk (Table 1) (1, 3, 7, 80–82, 161). For example, a meta-analysis by Li et al. demonstrated an elevated risk of CVD for both sexes with history of SLE, though this risk was disproportionately higher in women versus men (80). The Framingham Offspring study reported that young women with SLE were over 50 times more likely to suffer an MI versus those of similar age without history of SLE (13, 81, 162). Similarly, a meta-analysis by Aviña-Zubieta et al. reported a 50% increased risk of CVD mortality in women with RA when compared with the general population (82).

The link between systemic inflammatory disorders and CVD has been hypothesized to occur due to the pathological role that inflammation plays in the progression of atherosclerosis (1). Thus, these systemic rheumatologic conditions have been classified as risk-enhancing factors in the AHA/ACC 2018 Cholesterol Guidelines and should be considered for women during risk stratification and evaluation for statin initiation (3, 13, 163).

### 3.8 Mental and behavioral health

Many psychosocial, behavioral, and lifestyle factors have also been studied which disproportionally affect women and are strong risk factors for early-onset CVD (1, 13). Depression, for example, is 2-fold more common in women than men and is a recognized risk factor for incident MI and cardiac mortality, one study reporting a 9% attributable risk of acute MI from depression (Table 1) (13, 83, 164, 165). Current available research of other psychosocial factors which women have more exposure to including history of sexual and physical abuse, psychological stress, and post-traumatic stress disorder have also been postulated as strong risk factors for CVD (13, 166).

Unfortunately, the link between postpartum depression and anxiety for women during their childbearing years with future CVD risk has not been well studied and warrants future investigation (19). Likewise, additional research is needed to determine if addressing behavioral factors such as nutrition, stress, and exercise reduce a women's CVD risk, particularly women with history of APOs (16). Future clinical trials can investigate the efficacy of lifestyle interventions such as adopting a heart-healthy diet and regular physical activity in the prevention of future CVD (19).

## 4 Clinician’s guide to addressing current knowledge gaps and future directions

The appropriate risk stratification and prevention of CVD in women remain a significant challenge and a principal issue given the considerable burden of CVD in women (2, 8, 84, 167, 168). It is reported that only 42% of cardiologists felt adequately prepared to assess CVD risk in their female patients, with only 22% reporting using guideline-directed sex-specific guidelines (169).

### 4.1 Need for better risk sex-specific algorithms and risk assessment tools

Although current prevention guidelines have mentioned the inclusion of pregnancy history in the assessment of CVD risk, limited studies have emphasized the incorporation of pregnancy risk factors into predictive CVD scoring (16, 85). In fact, current CVD risk assessment tools do not consider any female-specific risk factors including APOs (19, 163). Only a few published studies have thoroughly investigated the utility of incorporating APOs to conventional CVD risk stratification despite their strong association with increased maternal CVD risk (19, 85, 92, 104, 170). This may be due to uncertainty as to whether APOs provide a direct causal relationship to future maternal CVD or if they unmask shared risk factors (16). For example, it is unclear if the delivery of SGA infants is an association independent of other maternal placental syndromes given their many interrelated factors.

Thus, further research is required to elucidate the true pathophysiology between these important sex-specific CVD risk factors with future maternal CVD risk to improve screening strategies, refine risk assessment, and implement primordial and primary prevention for women beyond traditional risk scoring algorithms (84). Future clinical trials and female-specific risk prediction models should recognize the importance of including women of childbearing age as well as women transitioning through menopause to reflect the target subpopulations intended for screening (16, 34).

### 4.2 Incorporating sex-specific questionnaires in patient evaluation

**Figure 1:**
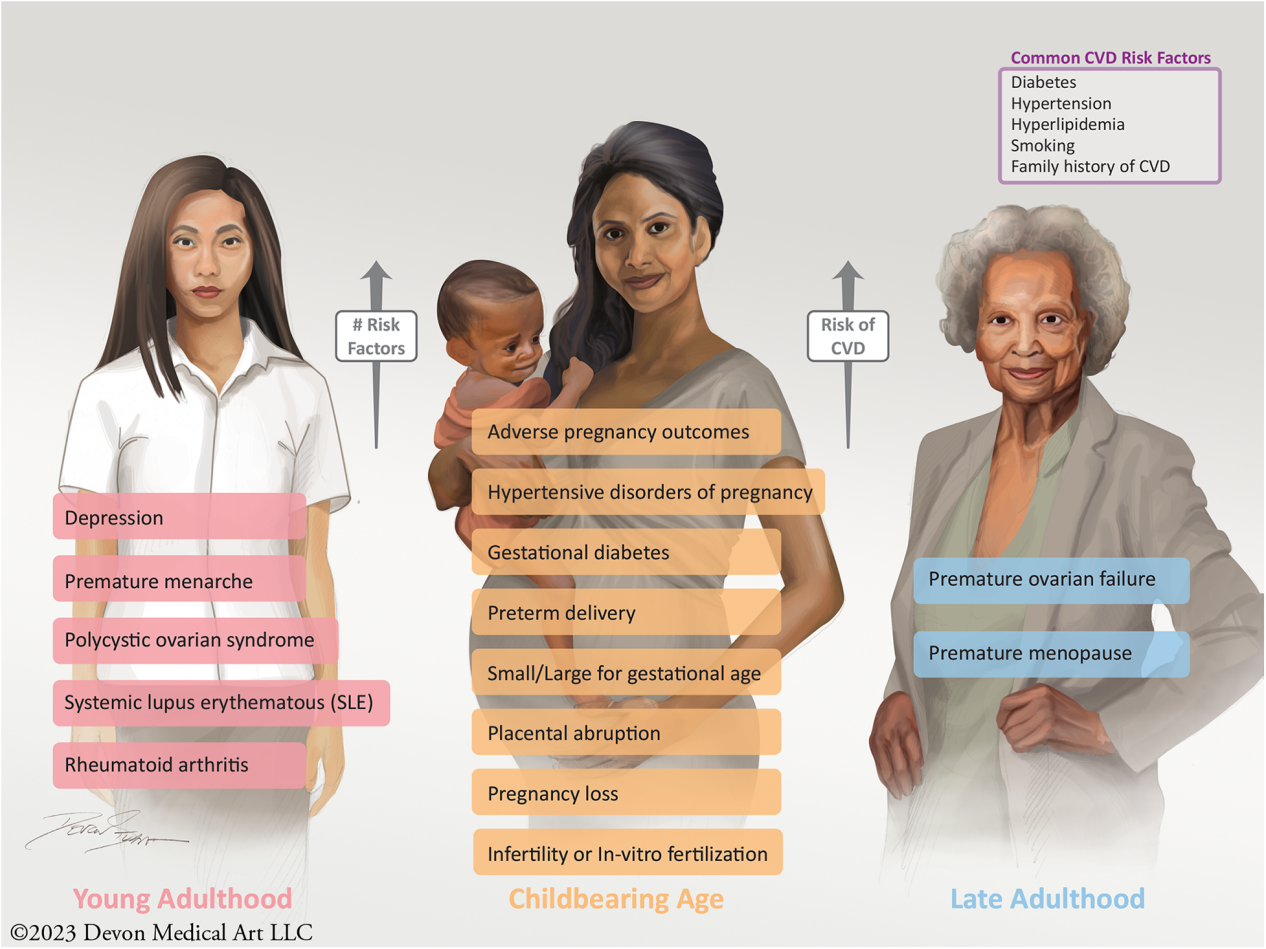
Sex-specific risk factors, which increase a women's future risk of CVD, can present over the span of a lifetime from young adulthood to childbearing age to late adulthood into retirement.

Improving patient and clinician education with regards to sex-specific CVD risk factors is vital. These risk factors can afflict women over a span of their lifetime, from young adulthood to childbearing age to their late adult and retirement years (Figure 1). Therefore, educating patients and clinicians, early and often, of these risk factors is essential to the identification and care of CVD in women. Most patients are not aware that having a pregnancy complication may increase their future CVD risk, with recent data showing that only 45% of women recognize that CVD is the leading cause of death (19, 169, 171). In particular, women with APOs should be informed that these disorders pose a higher lifetime risk of CVD and should undergo urgent risk assessment (19, 172, 173). Education and awareness of these risk factors have been shown to enhance the physician-patient relationship, improve engagement, and promote medication adherence (84, 174, 175). Likewise, educating clinicians and fellows-in-training regarding the importance of strong obstetrical and gynecological history-taking is fundamental and should be part of core and continuing medical education (84). Topics surrounding the identification of women with sex-specific risk factors should be featured at national and professional society conferences, such that all providers are better informed to provide comprehensive care for women at risk for CVD (84).

As evidenced by our discussion, a clinician's role in taking a strong obstetrical and reproductive history is an often neglected, though critical aspect, in the risk assessment and prevention of CVD in women. From preconception through pregnancy and into menopause, this continuum serves as an important opportunity for cardiovascular risk assessment. In fact, the American College of Obstetricians and Gynecologists (ACOG) recently formulated a concept called the “fourth trimester” of pregnancy, defined as a critical period for women after birth which warrant recurrent continuity of care beyond a traditional single postpartum visit (19, 171). With a multitude of elements of cardiovascular health to be discussed in a time-limited encounter, obtaining a strong sex-specific history poses a challenge (84).

**Figure 2:**
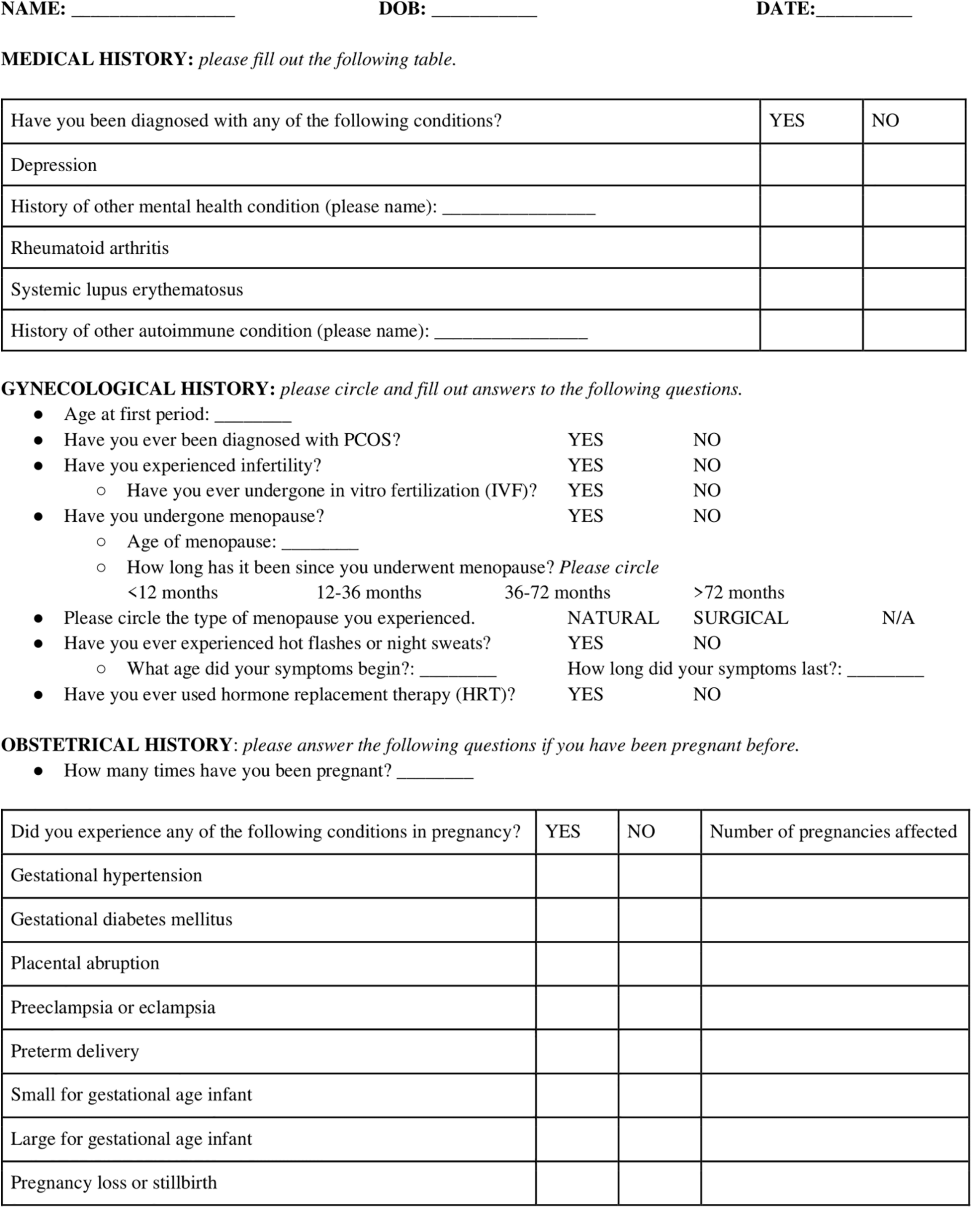
A screening questionnaire encapsulating pertinent medical, gynecologic, and obstetrical history to identify and document important sex-specific CVD risk factors.

To tackle this challenge, we developed a sex-specific screening questionnaire which can utilized and replicated throughout ambulatory clinics worldwide (Figure 2). This questionnaire highlights the many neglected sex-specific risk factors for women of reproductive age, which if recognized early, can assist in identifying high-risk individuals for close long-term follow-up and appropriate counseling regarding CVD prevention (Figure 2).

### 4.3 Prospective and longitudinal databases to study sex-specific risk factors

Although beyond the scope of this review, the following prospective registries and cohort studies have been instrumental in understanding sex-specific risk factors and its association with CVD: NuMo2B, WISE, SCAPIS, SWAN, and CARPREG II. Active enrollment of eligible patients into current registries and cohort studies is a necessary element to propel the investigation of sex-specific risk factors forward.

## 5 Conclusion

Cardiovascular care for women in our current standard of practice is far from ideal. As outlined in this review, obtaining a thorough obstetrical history represents an opportunity to encourage sex-specific risk factor screening and refine risk prediction and stratification of CVD by recognizing important aspects of a women's reproductive and obstetrical history which affect long-term cardiometabolic health (84). Incorporation of sex-specific risk factors is one important step in shifting the paradigm of underdiagnosing and undertreating CVD in women which traditional risk models have done for years (3, 84, 176). Implementation of our patient questionnaire is an efficient, large-scale, standardized method of eliciting important medical history as it pertains to sex-specific risk factors, and can be utilized as a data analysis tool to develop a future prognostic model to improve the current inadequate care of CVD in women.
